# Real-time whole-brain imaging of hemodynamics and oxygenation at micro-vessel resolution with ultrafast wide-field photoacoustic microscopy

**DOI:** 10.1038/s41377-022-00836-2

**Published:** 2022-05-17

**Authors:** Xiaoyi Zhu, Qiang Huang, Anthony DiSpirito, Tri Vu, Qiangzhou Rong, Xiaorui Peng, Huaxin Sheng, Xiling Shen, Qifa Zhou, Laiming Jiang, Ulrike Hoffmann, Junjie Yao

**Affiliations:** 1 Department of Biomedical Engineering, Duke University, Durham, NC 27708 USA; 2 Department of Pediatric Surgery, Second Affiliated Hospital of Xi’an Jiaotong University, Xi’an, Shaanxi China; 3 Roski Eye Institute, Department of Ophthalmology, Keck School of Medicine, University of Southern California, Los Angeles, CA 90033 USA; 4 Department of Biomedical Engineering, University of Southern California, Los Angeles, CA 90089 USA; 5 Department of Anesthesiology, Duke University, Durham, NC 27708 USA

**Keywords:** Biophotonics, Microscopy

## Abstract

High-speed high-resolution imaging of the whole-brain hemodynamics is critically important to facilitating neurovascular research. High imaging speed and image quality are crucial to visualizing real-time hemodynamics in complex brain vascular networks, and tracking fast pathophysiological activities at the microvessel level, which will enable advances in current queries in neurovascular and brain metabolism research, including stroke, dementia, and acute brain injury. Further, real-time imaging of oxygen saturation of hemoglobin (sO_2_) can capture fast-paced oxygen delivery dynamics, which is needed to solve pertinent questions in these fields and beyond. Here, we present a novel ultrafast functional photoacoustic microscopy (UFF-PAM) to image the whole-brain hemodynamics and oxygenation. UFF-PAM takes advantage of several key engineering innovations, including stimulated Raman scattering (SRS) based dual-wavelength laser excitation, water-immersible 12-facet-polygon scanner, high-sensitivity ultrasound transducer, and deep-learning-based image upsampling. A volumetric imaging rate of 2 Hz has been achieved over a field of view (FOV) of 11 × 7.5 × 1.5 mm^3^ with a high spatial resolution of ~10 μm. Using the UFF-PAM system, we have demonstrated proof-of-concept studies on the mouse brains in response to systemic hypoxia, sodium nitroprusside, and stroke. We observed the mouse brain’s fast morphological and functional changes over the entire cortex, including vasoconstriction, vasodilation, and deoxygenation. More interestingly, for the first time, with the whole-brain FOV and micro-vessel resolution, we captured the vasoconstriction and hypoxia simultaneously in the spreading depolarization (SD) wave. We expect the new imaging technology will provide a great potential for fundamental brain research under various pathological and physiological conditions.

## Abstract (web summary)

The ultrafast wide-field photoacoustic microscopy integrates novel light source, fast scanning mechanism, and machine learning enhancement, which allows high-speed tracking of whole-brain functions in action at capillary level.

## Graphical abstract

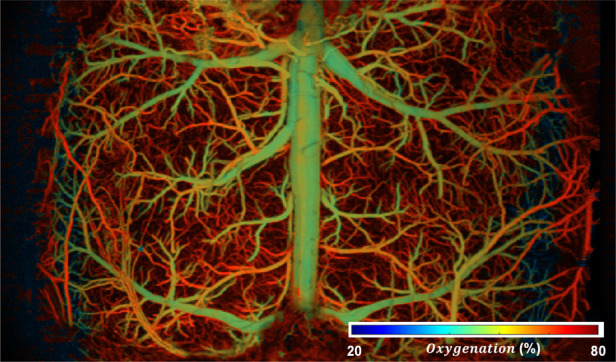

## Introduction

Imaging technologies capable of visualizing real-time hemodynamics in complex brain vascular networks, on a micro and macrolevel, are urgently needed to facilitate the understanding of current queries in neurovascular and brain metabolism research, including but not limited to stroke, dementia, and management of acute brain injury^1,2^. Further, additional features, like real-time oxygen saturation of hemoglobin (sO_2_) imaging, essential for the distinction of arteries and veins, are needed to capture fast-paced oxygen delivery dynamics and ultimately brain metabolism. However, advances in these fields have been hampered by the fact that existing brain imaging technologies suffer various limitations, particularly lacking imaging speed and image resolution as well as compromising the field of view (FOV).

While positron emission tomography (PET) and functional magnetic resonance imaging (fMRI) can provide excellent penetration, they suffer from low spatial and temporal resolutions. Optical microscopy is widely used for studying brain functions with high resolution, but it is often hampered by slow imaging speed^3^ or poor penetration depth^4^. Microbubble-enhanced ultrasound imaging has been used for brain research with deep penetration and high resolution, but it still lacks functional sensitivity^5^.

Photoacoustic microscopy (PAM), which detects ultrasound signals induced by optical absorption^6–8^, has demonstrated increasing impact in brain studies^9–12^. Particularly, PAM is capable of functional and molecular imaging with various endogenous and exogenous contrasts, such as measuring the blood oxygenation, blood flow, and metabolic rate of oxygen. However, like other scanning-based imaging technologies, it remains a technical challenge for PAM to achieve high imaging speed, large field of view (FOV), high spatial resolution, and high detection sensitivity simultaneously.

While traditional PAM systems use slow motor scanning^13,14^, recent advances in high-speed PAM have explored faster scanning mechanisms, including piezo scanner^11^, galvo scanners^15–17^, water-immersible microelectromechanical systems (MEMS) scanners^12,18–21^, and polygon scanning systems^22,23^. Among them, the polygon scanner can provide repeated line scanning over a large scanning range with simple rotary driving^24–26^. The polygon scanner is advantageous over the galvo scanner and MEMS scanner, because its scanning range does not depend on the scanning frequency.

Here, we present an ultrafast functional photoacoustic microscopy (UFF-PAM), which enables the imaging of whole-brain microvasculature and functional dynamics in response to physiological and pathophysiological challenges, with a wide FOV and high spatial resolution. We have developed a diamond-ground metal polygon with 12 facets, driven by a water-immersible high-speed DC motor, which can steer the confocal beam of laser excitation and ultrasound detection simultaneously. Its maximum line scanning rate is more than 2 kHz over an 11 mm scanning range, which is more than two times faster than the previously reported polygon PAM systems^27,28^. More importantly, we have developed dual-wavelength excitation at 532 nm and 558 nm, enabling functional brain imaging at high speed. We have developed an automatic image registration method to overcome the misalignment of the polygon facets due to water damping. Moreover, we have applied a deep-learning approach to mitigate the spatial undersampling and substantially improved the image quality.

As a proof-of-concept, we have demonstrated UFF-PAM of hemodynamic responses in mouse brains to hypoxia, sodium nitroprusside (SNP) induced systemic hypotension, and ischemic stroke. For the hypoxia challenge, UFF-PAM monitored the global hemoglobin deoxygenation in the brain and the resultant vasodilation. For the SNP challenge, UFF-PAM imaged the progress of arterial dilation and the resultant blood oxygenation dynamics. For the ischemic stroke, UFF-PAM captured the functional response of brain microvasculature during and post the stroke, particularly, the stroke-induced spreading depolarization (SD) waves. Enabled by the large FOV and high imaging speed, UFF-PAM can precisely pinpoint the SD wave’s originating position, and track its propagation direction and spreading pattern. With high spatial resolution, UFF-PAM can clearly resolve the local vasoconstriction and deoxygenation associated with the SD waves on the single-vessel level. All in all, with its unique capability of capturing fast hemodynamics, UFF-PAM may become a powerful tool to address a multitude of important questions in functional brain research.

## Results

### The UFF-PAM system

**Fig. 1:**
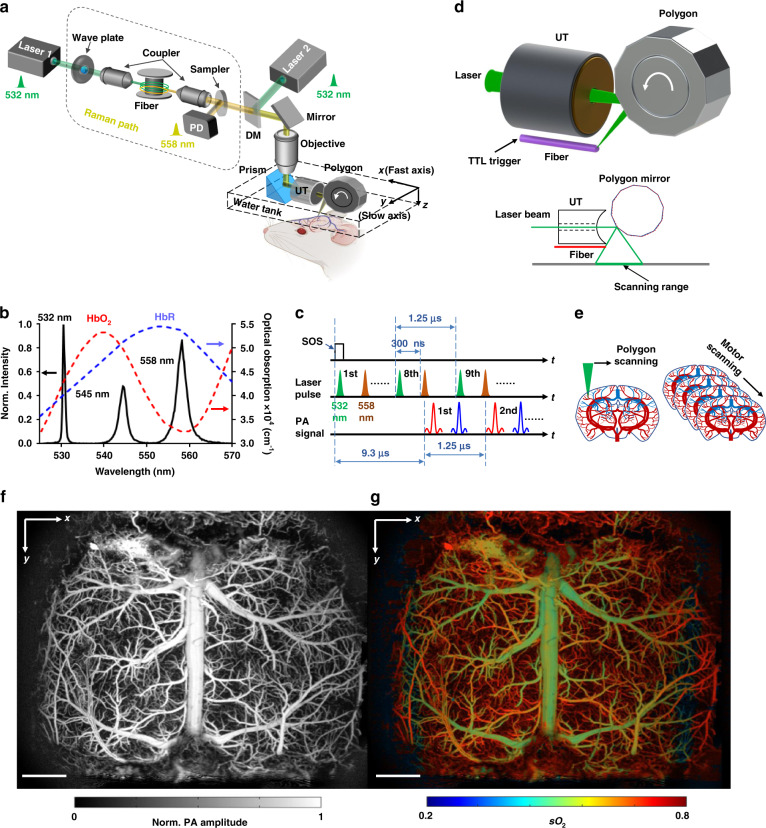
Ultrafast functional photoacoustic microscopy (UFF-PAM) with high imaging speed and wide field of view. **a** Schematic of the UFF-PAM system. The 532 nm light path and the 558 nm Raman path are combined for functional imaging. DM dichroic mirror, PD photodiode, UT ultrasound transducer. **b** The optical spectrum of the Raman path output, and the absorption spectra of oxyhemoglobin (HbO_2_) and deoxyhemoglobin (HbR). **c** The dual-wavelength excitation sequence and the resultant PA signals from HbO_2_ and HbR. **d** The close-up schematic of the ultrasound transducer, the polygon scanner, the start of scan (SOS) detection, and the scanning range. **e** Scheme of UFF-PAM scanning. Volumetric imaging is achieved by fast polygon scanning along the *x* axis (fast axis) and stepper motor scanning along the *y* axis (slow axis). **f** A representative *x–y* maximum amplitude projection (MAP) image of a mouse brain vasculature over the entire cortex, acquired by UFF-PAM at 532 nm. **g** The oxygen saturation of hemoglobin (sO_2_) map of the same mouse brain, acquired with dual-wavelength measurements at 532 nm and 558 nm. Scale bar in (**f**, **g**), 1 mm

Figure 1a illustrates the schematic of the UFF-PAM system. Two lasers are used for the dual-wavelength PA excitation (Supplementary Fig. S1a). One laser (VPFL-G-20, Spectra-Physics) generates the 532-nm light beam, and the other laser (SPFL-532-40, Spectra-Physics) is used for the 558 nm Raman path. A half-wave plate (AHWP05M-580, Thorlabs) adjusts the polarization state of the Raman pump beam to achieve a high Raman shift efficiency. The Raman pump beam is focused by an objective (UPLFLN 20×, Olympus) and then coupled into a 6.5-m-long polarization-maintaining fiber (HB450-SC, FIBERCORE) to generate the 558 nm light (Supplementary Fig. S1b). The conversion efficiency of the Raman path is about 33% at 558 nm (Fig. 1b). A dichroic mirror (DMSP550, Thorlabs) combines the 532-nm beam and the 558-nm beam. The combined laser beam is focused by an objective lens (AC127-050-A, Thorlabs) through the central aperture of a spherically focused ring-shaped ultrasound transducer (central frequency, 40 MHz; bandwidth, 100%; focal length, 14 mm). The optical beam spot size on the sample surface is ~10 μm. Both the excitation laser beam and the resultant PA waves are steered by a lab-made water-immersible 12-facet polygon scanner, with confocal alignment across the fast-scanning range (Supplementary Movie S1). The PA signal is amplified and sampled at 250 MHz (ATS9350, AlarzarTech). The laser firing, polygon scanning, stepper motor motion, and data sampling are all synchronized by an FPGA system (myRIO, National Instruments).

In UFF-PAM, the relative concentrations of oxyhemoglobin (HbO_2_) and deoxyhemoglobin (HbR) are quantified from the PA signals generated by the pair of laser pulses at 532 nm and 558 nm (Fig. 1c). A lab-made start of scan (SOS) detection system uses a multi-mode fiber to detect the starting position of the scanning laser beam by each facet (Fig. 1d and Supplementary Fig. S1c). While each laser pulse generates one-dimensional time-resolved PA signal, volumetric imaging is achieved by fast polygon scanning along the *x* axis (fast axis) and stepper motor scanning along the *y* axis (slow axis) (Fig. 1e). The SOS signal is used to synchronize the fast-axis polygon scanning with the slow-axis stepper motor scanning. Both the vascular structure and the oxygenation map can be obtained by a single scan with dual-wavelength excitation. No optical wavelength switching is needed. The PA signal is filtered by a lowpass filter (BLP-50 + , Mini-Circuits) with a cutoff frequency of 48 MHz before sampled by the DAQ card at 250 MHz (ATS9350, AlarzarTech). No signal averaging is performed. We have developed a longitudinal whole-cortex cranial window that is both optically and acoustically transparent for PA imaging (see “Materials and methods”). The cranial window can effectively improve the resolution and signal-to-noise ratio of the PA images, by eliminating the skull’s optical scattering and acoustic attenuation.

Figure 1f, g is the representative mouse brain images acquired by UFF-PAM, showing the whole-cortex vasculature network and vessel-by-vessel sO_2_ map, respectively. UFF-PAM can achieve a maximum time-resolved A-line frame rate of 2 MHz, a B-scan frame rate of 2 kHz over 11 × 1.5 mm^3^, and a C-scan frame rate of 2 Hz over an 11 × 7.5 mm^2^ FOV (Supplementary Fig. S2), which is 3600 times faster than the traditional slow-scanning PAM system. The FOV is sufficient to cover the entire cortex of an adult mouse brain. Supplementary Fig. S2a–h shows the brain vasculature images acquired with different imaging speeds, which essentially demonstrate the image quality at different spatial sampling densities. Slower imaging leads to denser spatial sampling and better spatial precision (Supplementary Fig. S2i–l). Supplementary Fig. S2m shows a maximum imaging depth of 1.5 mm achieved by the current imaging system using a single wavelength at 532 nm. Since the depth of focus is 0.42 mm, the spatial resolution becomes worse with the increasing imaging depth. In our experiments, the laser beam was approximately focused at 250 μm beneath the brain surface, and we estimate that lateral spatial resolution would degrade to ~127 μm at 1.0 mm depth due to the beam divergence and strong optical scattering. Please note that beyond 1 mm depth, the lateral spatial resolution of our system is mainly determined by the focused acoustic detection, i.e., the system effectively becomes an acoustic-resolution PAM or AR-PAM.

The imaging speed of UFF-PAM can be adjusted based on the laser pulse repetition rate (PRR) and the desired spatial sampling density (or scanning step size). With rotational scanning, the effective scanning step size along the fast axis is not uniform on the sample surface, with an increasing scanning step size toward the two ends of the scanning range (Supplementary Fig. S3a). In addition, limited by the laser PRR, there is a tradeoff between the B-scan frame rate and the scanning step size. Because the polygon scanner generates a curved in-focus scanning trajectory, the detection sensitivity and spatial resolution over the scanning range is not uniform, which is the highest in the middle and lowest on both ends (Supplementary Fig. S3b). The lateral resolution ranges from 7 µm to 18 µm over the scanning range (Supplementary Fig. S3c, d). Unlike the lateral resolution, the axial resolution of the UFF-PAM system is determined by the detected ultrasound bandwidth^29^, which is ~33 µm across the scanning range. For in vivo experiments, we usually reduce the volumetric frame rate to 0.3 Hz, with a PRR of 800 kHz, a B-scan frame rate of 1 kHz, and a scanning step size of 15–19 μm. Note that the relatively large scanning step size leads to severe spatial undersampling which will be addressed below.

### Facet registration and deep-learning-based upsampling

**Fig. 2:**
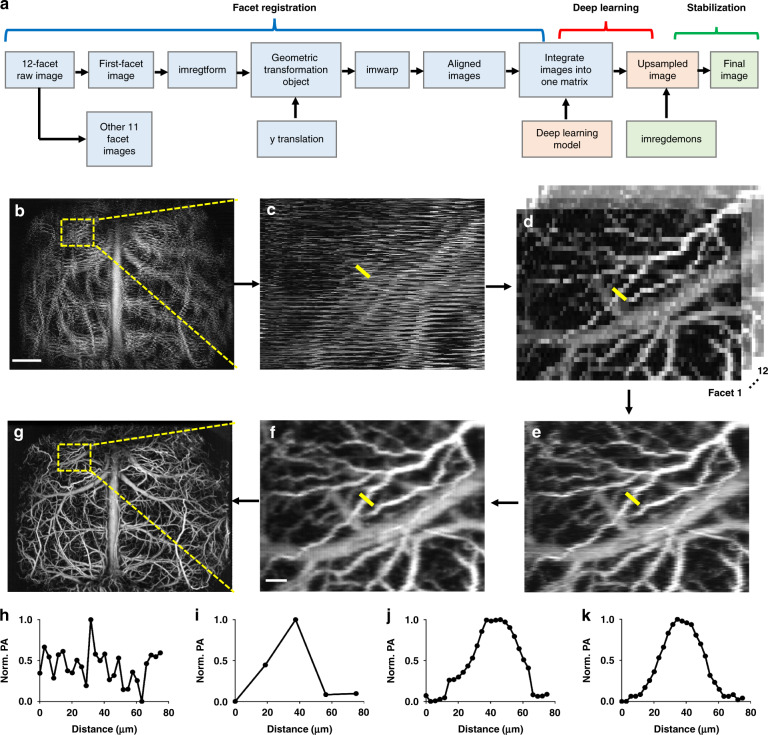
Image registration and deep-learning-based upsampling in UFF-PAM. **a** The flowchart of the imaging process in UFF-PAM, including the facet registration, deep-learning-based upsampling, and frame stabilization. **b** The misaligned and undersampled PA image of the whole cortex, generated by all 12 facets. **c** Close-up image of the dashed box region in (**b**), showing the misalignment of each facet. **d** Close-up images generated by every single facet. **e** Close-up PA image after fac*et al*ignment. **f** Close-up PA image generated by deep-learning-based upsampling. **g** The whole-cortex image improved by facet alignment and deep-learning upsampling. **h**–**k** The intensity profiles of the representative vessel marked by the yellow lines in (**c**–**f**), respectively. Scale bar in (**b**) and (**g**), 1 mm. Scale bar in (**c**–**f**), 100 μm

Figure 2a illustrates the data processing procedure in UFF-PAM, which includes facet registration, deep-learning-based upsampling, and frame stabilization. Because the polygon scanner is fully immersed in water for acoustic coupling, the water damping force induces the wobbling motion of the polygon. Moreover, the 12 facets have minor variations in their dimensions. Together, there exists misalignment among the scanning trajectory of different facets, resulting in discontinuous and distorted images (Fig. 2b, c). Although each facet can provide self-consistent scanning (Fig. 2d), the large scanning step size and undersampling lead to poor image quality. To mitigate the scanning misalignment due to polygon wobbling and facet variation, we have developed a geometric-transformation registration method to find the difference between each facet and subsequently realign the images (see “Materials and methods”). With image registration from all twelve facets, the misaligned image can be substantially improved with more continuous vessels (Fig. 2e).

However, even with the facets aligned, UFF-PAM still suffers from undersampling due to the large scanning step size. The undersampling results in discontinuous vessel boundaries and pixelized vessel lumen (Fig. 2e). To address the undersampling, we have developed a deep-learning-based upsampling strategy, adapted from our previous modified Fully Dense U-net (FD U-net) model upsampling pipeline (Supplementary Figs. S4 and S5; also see “Materials and methods”)^30^. We trained the upsampling model using fully sampled brain vasculature images to maintain vessel continuity, and used the trained model to restore the undersampled PAM images (Supplementary Fig. S4b). Compared with the undersampled image in Fig. 2e, the upsampled result in Fig. 2f shows smoother vessel boundaries, reduced undersampling artifacts, and more consistent vessel intensity and vessel profile (Fig. 2g). We also compared the deep-learning-based upsampled result with bicubic interpolation (Supplementary Fig. S6). Bicubic interpolation created images with blurring, jagged and discontinuous vessel profiles, and failed to improve the image quality. The facet alignment and image upsampling are critical steps to restore the vessel structures in UFF-PAM, providing better vessel fidelity for further quantitative analysis (Fig. 2h–k).

### In vivo high-speed functional imaging of cerebral hemodynamics by UFF-PAM

Imaging hemodynamics in the mouse brain in particular at the microvessel level is highly desired for understanding neurovascular coupling and the regulation of cerebral blood flow in both healthy and diseased states. To demonstrate UFF-PAM’s high speed and large FOV, we captured cerebral hemodynamic responses to three pathophysiological challenges: (1) systemic hypoxia, (2) systemic vasodilation with sodium nitroprusside (SNP), and (3) ischemia-triggered cortical spreading depolarization waves and spreading ischemia. The imaging parameters are summarized in Supplementary Tables S1 and S2.

#### Systemic hypoxia challenge

**Fig. 3:**
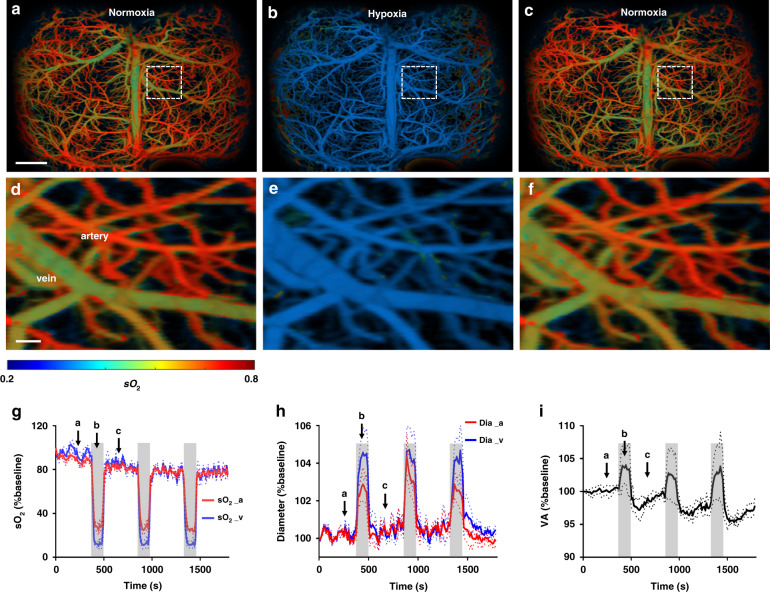
UFF-PAM of brain hemodynamics under hypoxia. **a**–**c** sO_2_ images of the entire mouse cortex under a cycle of normoxia, hypoxia, and then returned to normoxia. **d**–**f** Close-up images of the dashed box regions in (**a**–**c**). **g** The averaged sO_2_ changes in arteries (sO_2__a) and veins (sO_2__v). The gray areas indicate the hypoxia challenges. The arrows represent the time points corresponding to (**a**–**c**). **h** The diameter changes of the marked artery and vein in (**d**). **i** Vessel area (VA) change of the whole cortex. *N* = 4. The data in (**g**–**i**) are shown as mean ± s.e.m. Scale bar, 1 mm in (**a**–**c**), 100 μm in (**d**–**f**)

To demonstrate UFF-PAM’s performance in capturing fast hemodynamic responses in the brain, we applied systemic hypoxia to the animals by decreasing the inspiratory oxygen content from 21% (normoxia) to 3% (hypoxia). After baseline imaging under normoxia, we introduced hypoxia for 2 min and imaged the resultant hemodynamic changes in the brain vasculature. We repeated the normoxia–hypoxia cycle three times. At normoxia, the UFF-PAM images can clearly differentiate arteries and veins, based on their sO_2_ levels (Fig. 3a, d and Supplementary Movies S2 and S3). Upon the hypoxia challenge, we observed an immediate decrease in sO_2_ levels in both arteries and veins across the brain (Fig. 3b, e). By analyzing the arterial and venous vessels separately, we were able to depict their different changes in sO_2_, an important capability provided by the single-vessel resolution of UFF-PAM. As expected, the averaged sO_2_ level in veins decreased by 85 ± 2% (*P* < 0.001), while sO_2_ in arteries decreased by 70 ± 2% (*P* < 0.001), as shown in Fig. 3g. The reduction and recovery of sO_2_ levels were reproducible. Moreover, hypoxia induced significant vasodilation (Fig. 3h). Representative arteries dilated by 3% (*P* < 0.001), representative veins dilated by 4.5% (*P* < 0.001). By analyzing all vessels over the entire cortex, we found a clear increase in the total vessel area in the brain (Fig. 3i), confirming a well-documented phenomenon in humans^31–33^. Continuous PA imaging over a 30-min period further showed a gradual decrease in the baseline sO_2_ level even under the normoxia condition, likely due to the increase in oxygen extraction fraction (OEF) induced by the repeated hypoxia challenge, which was consistent with previous studies on humans under hypoxic conditions^34–36^.

#### Systemic vasodilation and vasoconstriction induced by sodium nitroprusside

**Fig. 4:**
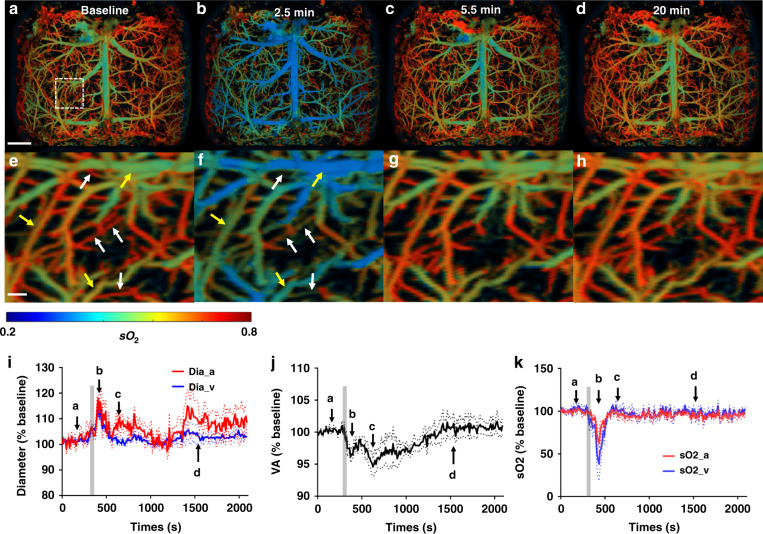
UFF-PAM of brain hemodynamics in response to SNP. **a**–**d** sO_2_ images of the entire cortex of the mouse brain at baseline, as well as 2.5, 5.5, and 20 min after SNP injection. **e**–**h** Close-up sO_2_ images at the respective time points, as indicated by the white dotted box in (**a**). The yellow arrows point to the representative dilated vessels, and the white arrows point to the representative constricted microvessels. **i** The average diameter changes of arteries and veins. The grey area represents the duration of SNP injection. **j** Vessel area change of the whole cortex. **k** The average sO_2_ change of arteries and veins. The arrows in (**i**–**k**) represent the time points corresponding to (**a**–**d**). *N* = 3. The data in (**i**–**k**) are shown as mean ± s.e.m. Scale bar, 1 mm for (**a**–**d**), and 100 μm for (**e**–**h**)

While the hypoxia experiment focused on physiological challenge-induced hemodynamic changes, the sodium nitroprusside (SNP) experiment aimed to investigate fast brain hemodynamics in response to a systemic hypotension. SNP is commonly used in clinical practice to dilate arteries and veins in order to ameliorate hypertension and reduce cardiac afterload^37–40^. We used UFF-PAM to detect fast hemodynamic changes induced by systemic SNP administration (Supplementary Movies S4 and S5). After monitoring the brain for 5 min as the baseline (Fig. 4a, e), a single dose of SNP was infused via a femoral vein catheter over 40 s. We continuously monitored the hemodynamic response in the brain for 35 min. As expected, SNP induced substantial vasodilation and thus a decrease in systemic blood pressure from 80 mmHg to 30 mmHg, measured by an intravascular pressure sensor (Supplementary Fig. S7). UFF-PAM observed the vasodilation process in the brain, which peaked at 2.5 min in major arteries and veins after the SPN infusion, with an 18 ± 2% (*P* < 0.001) increase in the vessel diameters (Fig. 4b, f). Vasodilation was more pronounced in major arteries (19 ± 5%) than in major veins (15 ± 2%) (Fig. 4i). Consistent with SNP’s short effective duration, the venous dilation gradually subsided after about 5 min, while the arterial dilation persisted longer.

Counter-intuitively, we also observed clear vasoconstriction in many microvessels (Fig. 4e, f). In fact, the vasoconstriction of the microvessels outweighed the vasodilation of the major vessels, and thus the total vessel area deceased by as much as ~5% (Fig. 4j). We believe that the SNP-induced decrease in the systemic blood pressure (Supplementary Fig. S7) constitutes the major reason for the observed microvascular constriction. Overall, SNP caused stronger cerebral vessel dilation than systemic hypoxia, especially in the arteries, consistent with its pharmacological profile and clinical use^40^. Drastically different responses to SNP in various vessel types highlighted the value of large FOV and high resolution of UFF-PAM.

We further observed significant deoxygenation of hemoglobin after SNP administration. The venous sO_2_ decrease (65 ± 10%, *P* < 0.001) was much larger than the arterial sO_2_ decrease (38 ± 4%, *P* < 0.001), within 2.5 min after the SNP administration (Fig. 4k). The different arterial and venous responses may reflect that SNP affected both oxygen delivery and consumption. Further, as shown in Supplementary Fig. S7, brain tissue oxygenation decreased substantially in tandem with the decrease in systemic blood pressure. Similar to the microvessel constriction in the brain, the constricted microvessels in the lung tissues might reduce the gas exchange efficiency and thus lead to a decrease in the arterial sO_2_^41,42^. Reduced capillary perfusion resulted in a higher oxygen extraction fraction (OEF), which contributed to the lower sO_2_ in the collecting veins (i.e., higher oxygen consumption) and a decrease in tissue oxygenation^39^. Overall, with its high speed, large FOV, and high resolution, UFF-PAM not only confirmed the expected global vasodilation induced by SNP but also observed the vasoconstriction in microvessels and global deoxygenation.

#### Stroke-induced cortical spreading ischemia

Spreading depolarization (SD) waves are intense neuronal and glial depolarization waves that propagate at a speed of millimeters minute^−1^ in the gray matter and are associated with massive transmembrane ion and water transportation, elevated extracellular excitatory amino acid levels, and a heavy metabolic burden to homeostasis^43,44^. Recurring SD waves can originate from the ischemic tissue in a spontaneous and random manner, and propagate throughout the vulnerable hemisphere^45,46^. The peri-infarct SD waves can exacerbate ischemic injury by imposing an additional metabolic burden with vasoconstriction, also termed spreading ischemia^44,47,48^. In addition, it has recently been reported that the lack of oxygen supply in the brain tissue can trigger SD waves^49^. In this experiment, we aimed to study the propagating cortical SD waves and the associated hemodynamics at the microvessel level. To do so, we performed a permanent stroke procedure on the left hemisphere by completely occluding the left carotid artery (Supplementary Fig. S8). We also temporarily occluded the right carotid artery to increase the ischemic burden and reduce the collateral blood supply through the circle of Willis. We used UFF-PAM to monitor the cortical vasculature and hemoglobin oxygenation changes throughout the entire procedure (Supplementary Fig. S9 and Supplementary Movies S6 and S7).

**Fig. 5:**
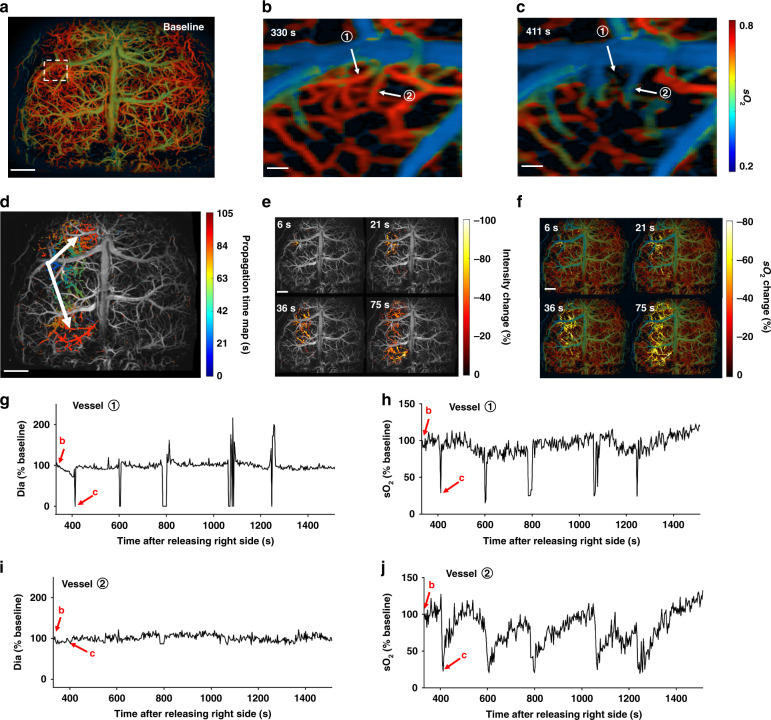
UFF-PAM of the stroke-induced SD waves. **a** sO_2_ image of the whole cortex at baseline. **b**, **c** Close-up sO_2_ images of the SD wave origin at 330 s and 411 s after releasing the right carotid artery, as indicated by the white dotted rectangle in (**a**). Two representative vessels ① and ② were selected for further analysis. **d** Propagation time map of a representative SD wave. Arrows represent the direction of SD wave. **e** The change in PA signal intensity during the SD wave propagation at 6 s, 21 s, 36 s and 75 s, respectively. **f** The change in sO_2_ during the SD wave propagation. **g** The diameter change of vessel ① over a SD wave. **h** The sO_2_ change of vessel ① over a SD wave. **i** The diameter change of vessel ②. **j** The sO_2_ change of vessel ②. Scale bars, 1 mm for (**a**, **d**, **e**, **f**), 100 μm for (**b**, **c**)

After ~9 min of bilateral carotid artery occlusion, we observed five spontaneous SD waves in the permanently occluded left hemisphere (Supplementary Movies S8 and S9). There are several important observations by UFF-PAM enabled by its large FOV, high speed, and high resolution. (1) By tracking the wave propagation over time across the left hemisphere, we were able to calculate the average SD wave speed as 2.56 ± 0.23 mm min^−1^, which was consistent with previous reports^50–52^. (2) We were able to precisely localize the originating point of each SD wave (Fig. 5b, c and Supplementary Movie S10), and map the spreading direction, pattern, duration, and affected area (Fig. 5d). This information was critical in mapping the core and penumbra areas in the cerebral stroke. (3) We were able to quantify the local vasoconstriction along the propagation path of an SD wave (Fig. 5e, g, i), which were followed by the decrease of local sO_2_ (Fig. 5f, h, j). Our results confirmed that, along the SD wave path, the local oxygen demand of the peri-infarct tissue could not be matched by the compensatory increase in local blood supply, leading to an increase in the oxygen extraction fraction. As a result, our results have clearly shown that the SD waves were essentially spreading waves of vasoconstriction, accompanied by spreading waves of hypoxia, which effectively demonstrated the spreading ischemia. UFF-PAM can provide the imaging speed, resolution, and FOV needed to capture the vasculature morphology and oxygenation changes during an SD wave event, which to the best of our knowledge has not been achieved previously by other photoacoustic imaging technologies.

## Discussion

In conclusion, we have developed an innovative UFF-PAM system that has simultaneously achieved a high imaging speed, large field of view, and high spatial resolution. By using stimulated Raman scattering (SRS)-based dual-wavelength exaction and water-immersible polygon-scanner, UFF-PAM can capture the hemodynamic changes in the brain, with a C-scan frame rate of 2 Hz over an FOV of 11 × 10 mm^3^, and a lateral resolution of 10 μm. The maximum B-scan frame rate is more than 2 kHz over a 11-mm scanning range. To our best knowledge, UFF-PAM is currently the fastest functional PAM system, while our previously reported polygon PAM system was not capable of functional imaging^28^. An automatic imaging registration method and a deep-learning-based upsampling approach were developed to mitigate the inter-facet misalignment and the spatial undersampling.

When fast scanning is needed, a rotating polygon mirror is a popular choice due to its simplicity and high speed. There is no need for an oscillating component or a complex driving system. Unlike the commonly used galvo scanners, which allow flexible control of the beam position, the maximum scanning angle of the polygon scanner is determined by the number of facets and the incoming beam size. We developed a water-immersed polygon scanner and calibrated for various misalignment between different facets. However, in our current setup, the need for water immersion limits the maximum imaging speed and image quality, mostly due to the water’s damping force and resistance. Polygon scanners operating in air are more stable and faster than water-immersible polygon scanners. Cylindrically focused ultrasound transducers coupled with fast optical scanning can be utilized to mitigate the need for scanning the acoustic beam.

Compared to the piezo-scanner-based system^11^, the current polygon-based system is about ten times faster. Compared with our pervious high-speed PAM using a water-immersible MEMS scanner^12^, the current polygon-based system is about four-times faster with a three-times larger scanning range. Previously, we reported the first implementation of high-speed PAM based on a 6-facet polygon scanner^28^, which, however, was not capable of functional imaging with a single-wavelength excitation. Moreover, because of the limited laser pulse repletion rate, the image quality was compromised by spatial undersampling. Compared to our previous work, the current polygon-based system has achieved functional imaging with a higher speed and better image quality. Compared to the previously reported polygon-based system with dual-wavelength excitation^27^, the maximum line scanning rate of our system is more than two times higher. The facet co-registration and upsampling are also critical innovations to improve the image quality.

Because of the fast imaging speed, limited laser pulse rate and the laser safety limit, UFF-PAM has severe undersampling along the fast-scanning axis. This undersampling cannot be addressed by using interpolation methods, which can introduce image blurring and aliasing artifacts, such as jagged vessel boundaries, and cannot effectively improve the resolution. To tackle this problem, we applied a deep-learning model modified from the Fully Dense U-net (FD U-net) to upsample the original UFF-PAM images, which builds upon the popular U-Net architecture using Dense blocks and robust regularization in the form of 2D spatial dropout. This spatial dropout randomly drops entire convolutional filters during model training, thereby ensuring that both feature redundancy and robustness are incorporated into our model. This model was trained to reconstruct undersampled UFF-PAM images of in vivo mouse brain vasculature with as few as 2% of the original pixels^30^. In addition, because our model learns to restore vessel continuity, it can also remove any remaining facet misalignment. With the deep-learning-based upsampling, the final UFF-PAM quality is similar to that with full sampling density (Fig. 2 and Supplementary Fig. S2). Therefore, the quantitative precision of the vascular morphology such as vessel diameter and area should be mainly determined by the spatial resolution of the system.

There are two timescales related to our imaging and data acquisition process: (1) the signal traveling time from the target to the ultrasound transducer, and (2) the data acquisition time that determines the imaged tissue thickness. First, it needs to be clarified that the time interval between consecutive A-lines (laser pulses) is shorter than the traveling time of the PA signals from the target to the ultrasound transducer. Therefore, we do not need to wait for the corresponding PA signals to arrive at the transducer before we fire the next laser pulse. We carefully designed our triggering sequence of the laser firing and the data acquisition so that we don’t miss or mix the PA signals from different laser pulses. Second, the time interval between the two-wavelength excitation laser pulses in our in vivo experiments was 300 ns, which effectively determined the imaged tissue thickness to be 0.45 mm with a speed of sound of ~1500 m s^−1^. We empirically chose this time interval mainly because the depth of focus of our focusing objective lens is ~0.42 mm. Outside the depth of focus, the PA signals are weak and the spatial resolution degrades. Nevertheless, this time interval can be changed, according to the imaged tissue thickness.

The 300-ns delay between the two laser pulses is much shorter than the thermal diffusion time of the first excitation pulse at 532 nm, and there might be a heating effect that could lead to elevated PA signal from the second excitation pulse at 558 nm, a phenomenon called the “Grüneisen-relaxation effect”^53^. It is a complex process that depends on the expiation pulse energy, the effective spatial resolution, the imaging depth, the optical absorption of the target, the thermal diffusion coefficient, and the time interval between the two laser pulses. While it is challenging to experimentally validate the thermal impact in vivo, we have estimated that the temperature rise on the tissue surface due to the first laser pulse is about 3.3 °C. This temperature rise can potentially increase the PA signals of the second wavelength by a maximum of 13%, which may lead to a systematic error in the sO_2_ quantification. Reducing the first laser pulse energy or increasing the time interval between the two laser pulses can effectively reduce such impact. Nevertheless, dual-pulse excitation has been widely used in photoacoustic imaging of blood oxygenation^27,54–57^, and its accuracy has been validated by previous studies. The impact of the dual-pulse time interval on quantifying blood oxygenation needs further investigation.

Besides, in our current system setup, the distance between the polygon facet surface and the target is about 9 mm, which requires 6 μs for the PA signal to travel. We have estimated that during the PA signal travel time, the polygon rotates by 0.18°, which effectively shifts the acoustic focus of the ultrasound transducer by ~28 μm. Since the ultrasound transducer has a focal spot size of ~127 μm, assuming the acoustic focus follows a Gaussian profile, the detection sensitivity of the ultrasound transducer is reduced by ~12.6% when the focus is shifted by ~28 μm. Since such sensitivity reduction is the same for both excitation wavelengths, the oxygenation quantification is not impacted.

Using UFF-PAM, we can image fast hemodynamic pathophysiology in the mouse brain on the global and local level, and can investigate oxygen delivery versus extraction consumption^−1^ dynamics based on the hemoglobin oxygenation measurement. As our hypoxia results demonstrate, UFF-PAM can make a unique contribution to the understanding of arterial oxygen content-induced changes in cerebral blood flow (CBF) regulation. While the mechanisms underlying the influence of hypoxia upon CBF are complex and involve interactions of many physiological, metabolic, and biochemical processes, there is emerging evidence showing that de-oxy-hemoglobin is the primary regulator of CBF^33^. We have shown that during hypoxic conditions arterial and venous sO_2_ is affected differently, more pronounced in veins, as OEF increases. Therefore UFF-PAM not only allows to observe global hemoglobin oxygenation kinetics as other technologies, but also to differentiate between arteries and veins, which reflect oxygen delivery versus oxygen extraction or consumption. Applying our system on a well-known hypoxia model^33^, our results support the important role of sO_2_ in the regulation of blood flow. Next, we investigated the cerebral response, with particular focus on the microcirculation, to critical hypotension induced by the systemic vasodilator SNP. SNP has been widely used in clinical practice as a vasodilator (e.g., during the hypertensive crisis) for more than 50 years^58–62^. However, studies of SNP effects on the brain vasculature have remained elusive^38,40^. Although our previous study reported the brain hemodynamic response to SNP in vivo^63^, and found vasodilation and decreased oxygenation after SNP application, we were not able to capture the functional dynamics in different types of vessels, due to the low imaging speed (3 min per image) and small FOV (3 × 3 mm^2^). By contrast, UFF-PAM was capable of visualizing the rapid hemodynamic response to SNP-induced substantial hypotension. Our results have demonstrated the rapid vasodilation in large vessels and yet constriction in microvessels, further underlining the necessity for high-resolution imaging of blood oxygenation. Lastly, our spreading ischemia experiments demonstrated that prolonged cerebral ischemia could trigger microvascular constriction waves, resulting in increased oxygen extraction. These microvascular constriction waves follow spreading depolarization events in the brain^44^, which have been proved to contribute to secondary lesion enlargement. The high-resolution and large FOV of UFF-PAM allowed monitoring the local initiation of the SD, as well as tracking the propagation pattern and vasoconstriction over the entire event across the whole cortex. UFF-PAM can potentially be used for the stroke research community to discern stroke core, penumbra, as well as expansion of the penumbra in real time, and assess neuroprotective therapies in future studies.

In summary, we have developed a high-speed wide-field functional PAM technology that may provide a powerful tool for studying the cerebral hemodynamics of the mouse brain in a wide range of pathological and physiological models.

## Materials and methods

### Animal preparation

All animal procedures were reviewed and approved by the Institutional Animal Care and Use Committee (IACUC) of Duke University (Protocol No.: A009-20-01), and were conducted in accordance with National Institutes of Health guidelines. C57BL/6 mice (3–5 months old; weight, 20–30 g) were used in all the in vivo experiments. The pulse energy at the surface of the mouse brain was ∼200 nJ per wavelength. The optical beam was focused 250 μm deep beneath the tissue surface. A 3D-printed nose cone securely mounted the mouse head. We kept the mouse under anesthesia with 1.5% v/v isoflurane throughout the imaging experiment, and the body temperature was maintained at 37 °C using a heating pad.

### The 12-facet polygon scanner

The 12-facet polygon scanner was fabricated from aluminum, and single-point diamond machining technology was applied to create the optical facets. Its clear aperture is 5 mm along the fast-scanning axis and 9 mm along the slow-scanning axis. The polygon diameter is 21 mm, and light reflection on each facet is >88% at 532 nm. A water-immersible DC electric motor (M1N10FB11G, Minebea Mitsumi) enables the polygon to operate smoothly at speeds ranging from a few rpm (revolutions per minute) to greater than 10,000 rpm, which corresponds to more than 2 kHz line scanning rate for the 12-facet polygon (Supplementary Movie S1).

### The ring-shaped ultrasound transducer

A ring-shaped, focused ultrasound transducer with a central frequency at 40 MHz was designed and fabricated in the lab. First, a piezoelectric transducer modeling software (PiezoCAD) based on Krimboltz, Leedom, and Mattaei (KLM) equivalent circuit model was employed to optimize the design of the transducer. According to the optimized parameters, a lithium niobate (LNO) plate (70-μm thickness) (Boston Piezo-Optics) was selected as the core piezoelectric layer. Next, a 10-μm-thick first matching layer made of silver-loaded epoxy and a 3-mm-thick backing material (conductive silver paste, E-solder 3022, Von Roll Isola) were deposited onto the front and back sides of the LNO plate, respectively. The matched and backed acoustic stack was then machined into a ring and inserted and fixed into a brass housing with a height of 4 mm. A central aperture with a diameter of 3 mm was drilled to deliver light, enabling co-axial alignment of optical excitation and acoustic detection for maximum detection sensitivity. The outer diameter of the transducer element is 6 mm. The gap was filled with epoxy resin (EPO-TEK 301, Epoxy Technology) and one lead wire was connected through the backing. After that, the transducer was pressed by a metal ball to form a focal length of 8 mm. A Cr/Au (50/100 nm) electrode was sputtered across the first matching layer and the brass housing to form a common ground connection. Finally, a 10-μm-thick parylene layer was deposited onto the entire external surface of the device as the second matching layer to compensate for the acoustic impedance mismatch with water.

### The start of scan (SOS) system

We used the PA excitation light as the laser source and an optical fiber as the receiver to build the lab-made SOS system. A multimode fiber (M45L02, Thorlabs) was mounted beneath the ultrasound transducer to receive the laser light. When the polygon rotated to the starting edge of each facet, the steered laser beam landed on the tip of the multimode fiber. A high-speed photodiode (PDA36A2, Thorlabs) was used to detect the received light by the fiber, and converted it into a SOS trigger signal. The lab-made SOS system was compact with high accuracy. The SOS signal triggered the FPGA card which synchronized the two PA lasers and the DAQ card.

### The SRS based dual-wavelength laser system

The 6.5-meter polarization-maintaining single-mode fiber (HB450-SC, FIBERCORE) was positioned with a fiber alignment stage (561D-XYZ, Newport) and a bare fiber chuck (FPH-S, Newport). The fiber tip was fixed with a universal bare fiber terminator (BFT1, Thorlabs), and the output beam from the fiber was collimated by an achromatic fiber port (PAF2A-A10A, Thorlabs). We measured the optical spectrum of the output laser after the achromatic fiber port with an optical spectrometer (CCS175, Thorlabs). The critical power and the maximum power of the *n*th-order Raman amplification in a silica fiber are given by^64^

1 $$P_N^{Cr} \approx 16 \times N \times A_{eff}/\left( {g_RL_{eff}} \right)$$

2 $$P_N^{Max} \approx 30 \times N \times A_{eff2}/(g_{R2}L_{eff2}){{{\mathrm{exp}}}}( - \alpha L)$$

where $P_N^{Cr}$ and $P_N^{Max}$ are the necessary pumping power to reach the Raman threshold and the maximum output power of the *n*^th^-order Stokes, respectively. The $A_{eff}$ and the $A_{eff2}$ are the effective mode area of the pump light in the fiber for the first and second stokes waves, respectively. $g_R$ and $g_{R2}$ are the Raman gain coefficients.$L_{eff}$ and $L_{eff2}$ are the effective lengths for first and second stokes waves, respectively. *L* is the fiber length, and α is the fiber attenuation coefficient. The maximum Raman scattering efficiency of the single-mode fiber occurs at a frequency shift of ∼13.2 THz, corresponding to 545 nm for the first stoke and 558 nm for the second stoke. We chose the second stoke light for functional imaging because of the larger difference in the optical absorption between oxy- and deoxyhemoglobin. The 558 nm light pulse energy after the dichroic mirror (DMSP550, Thorlabs) was up to ~500 nJ.

### Inter-facet image registration

Image registration is used to find an optimal spatial transform that maps pixels from the template image *I*_1_ by the reference facet to the corresponding pixels in the target image *I*_2_ by the misagligned facet,

3 $$I_1 = \left[ {x\,y} \right];\,I_2 = \left[ {x^\prime \,y^\prime } \right]$$

The target image *I*_2_ has a distortion relative to the template image *I*_1_ that can be described by a 2D geometric transformation *T*,

4 $$\left[ {x^\prime \,y^\prime \,1} \right] = [x\,y\,1] \times T$$

5 $$T = \left[ {\begin{array}{*{20}{c}} {s_c} & { - s_s} & 0 \\ {s_s} & {s_c} & 0 \\ {t_x} & {t_y} & 1 \end{array}} \right]$$

where *T* is a 3-by-3 matrix that depends on four parameters.

6 $$s_c = scale \times {{{\mathrm{cos}}}}(angle)$$

7 $$s_s = scale \times {{{\mathrm{sin}}}}(angle)$$

where “scale” is the scaling factor, “angle” is the rotation angle, *t*_*x*_ is the *x*-translation, and *t*_*y*_ is the *y*-translation. We chose the image from one facet as the template (reference) image, and the other 11 images were used as the target images. After extracting the corresponding geometric transformation *T*, we performed the inverse transform T^−1^ on the 11 target images. A simple automated image registration method was used to address the misalignment between the twelve facets. There were five steps in the registration process.

Step 1. We extracted every single-facet image from the entire volume scan. We chose the first facet image as the fixed image known as I_1_ and the images from the other 11 facets as the moving images known as I_2_. The adjacent B-scans from every single-facet image were aligned in the raw data.

Step 2. We used the function “imregtform” in MATLAB to find the geometric transformations that mapped the images to be registered (moving) to the reference image (fixed). A “similarity” model was used, which included translation, scaling, and rotation.

Step 3. We modified the geometric transformation and added *y*-translation, since there was *y* axis shift between each facet.

8 $$t_y = t_{y^\prime } - (i - 1)/12$$

where *t*_*y*’_ is the *y*-translation calculated from the “imregtform”, *i* is the *i*th facet, and *t*_*y*_ is the modified *y*-translation.

Step 4. We performed the transformation by using the “imwarp” function, which used the geometric transformation to map coordinates in the output image to the corresponding coordinates in the input image (inverse mapping).

Step 5. We interleaved lines from each facet into one final composite image.

### Deep-learning model for image upsampling

As described in our previous work^25^, we tested various deep-learning model architectures, including U-net and some variations on U-net, and found a modified FD U-net performed best at the task of upsampling PAM images (see Supplementary Fig. S4a for model depiction). The original FD U-net improved upon the traditional U-net architecture by replacing the typical convolutional blocks with Dense blocks—which had previously been shown to improve the performance of deeper convolutional neural networks (see Supplementary Fig. S4b for an in-depth depiction of Dense blocks). We then further modified this FD U-net archetype by replacing RELU activation functions with ELU (which also helps improve the performance of deeper networks), replacing max pooling with strided convolutions (to allow for learned downsampling within the network), and by adding spatial dropout (*P* = 0.05) to regularize the network and improve robustness. This last modification, in addition to the extensive image augmentation used during training, are especially important in this work, as a model originally trained on traditional PAM images was able to successfully extrapolate to 12-facet polygon PAM images. Due to updates in the deep-learning framework used (i.e., Tensorflow) we were able to discard the “model patchwork algorithm” of our previous work—which processed 128 by 128 pixel crops of larger images—in favor of applying our fully convolutional neural network on entire 12-facet images at a time. The general deep-learning image processing framework is depicted in Supplementary Fig. S5 and went as follows:

Step 1. We saved the mins and maxes of the facet-aligned polygon images and normalized the images to between [0, 1].

Step 2. We performed an initial upsampling to change the images to their final upsampled shape by adding zero pixels to represent where undersampling occurred (see Supplementary Fig. S5*Zerofill Image*).

Step 3. We fed these images as input into our deep-learning model. Although the model was originally trained on 128 × 128 pixel crops, larger images can now be used as input during inference (as our neural network is fully convolutional).

Step 4. We then renormalized each of the images using the saved mins and maxes, to allow for extraction of functional information (such as sO_2_).

### Transcranial brain window

To optimize the in vivo performance, we developed a whole-cortex transcranial brain window that was both optically and acoustically transparent. The window was trapezoidal-shaped, with a 9 mm by 10 mm frame size and a useable opening of 8 mm by 7.5 mm. To avoid compressing brain tissue and damaging blood vessels, the window frame had a curved surface that followed the mouse skull’s natural curvature. The window frame was 3D-printed using PLA. The window opening was sealed with PVC membrane (10-μm thick) that was both optically and acoustically transparent.

To install the brian window, mice were anesthetized by 100 mg kg^−1^ ketamine and 10 mg kg^−1^ xylazine. After mounting the mouse head into a stereotactic frame, mice were shaved, and the head surface was cleaned using iodine and alcohol. After midline skin incision, the skin was retracted to fully expose the skull from the olfactory tract area to the occipital area and laterally to the border of the temporal muscles. The skull was kept wet using saline, and two coronal lines at the level of AP −2 mm and AP + 4 mm and two sagittal lines along the border of temporal muscles were drilled until the skull became moveable. Using bone wax to seal tiny bleeding sites, the skull was carefully lifted and removed. The transcranial window was then mounted on the skull and glued using Cyanoacrylate (BSI, Atascadero, CA). Next, the skin was glued along the side edges of the window frame. The mouse was returned to the home cage and treated with three days of 5 mg kg^−1^ carprofen via subcutaneous (s.c.) injection (Levafen injection, Patterson Veterinary) and seven days of 5 mg kg^−1^ enrofloxacin via s.c. injection (Sigma). After a recovery period of 7 days, mice were ready for imaging.

### Systemic hypoxia

Mice were anesthetized with 1.5% v/v isoflurane and kept on a heating pad to maintain body temperature at 37 °C. Systemic hypoxia was induced by decreasing the oxygen content of the inspiratory air from 21 to 3% via flowmeters. After normoxia baseline imaging for 6 min, we introduced hypoxia for 2 min and imaged the resultant physiological changes. We repeated the normoxia/hypoxia cycle for three times to test the stability of the system. We monitored the mice continuously over 30 min.

### SNP application

SNP solution (Somerset Pharma) was prepared in saline (concentration: 250 μg mL^−1^) immediately before use, and kept in the dark environment at 4 °C to prevent deterioration. SNP (25 mg kg^−1^) was injected via a femoral vein catheter (PE 10) over a 40 s period using a syringe pump. In a subset of mice, we also cannulated the femoral artery and measured systemic blood pressure and brain tissue oxygenation. For monitoring ptO_2_ in the cortex, we used an oxygen electrode (Clarke-style, 25-micrometer tip diameter; Unisense, Picoammeter PA 2000, Arhus) over the observation period.

### Cerebral ischemic stroke

The left carotid artery was permanently ligated with a 6.0 suture, while the right carotid artery was clamped temporally with a small vascular clip. After 9 min of dual carotid artery occlusion, the clamp on the right carotid artery was removed to allow restoration of blood flow and reperfusion.

### PA data analysis

We carry out quantitative data analyses based on the imaging results to evaluate cerebral hemodynamics and metabolism changes corresponding to physiological states (hypoxia challenge, SNP, and ischemic stroke). By using the lab-developed vessel segmentation method, we quantified the total vessel area, average sO_2_ (a threshold of 0.65 to distinguish arteries from veins), and averaged vessel diameter. A total of 6–8 arteries and 6–8 veins per mouse were analyzed, with vessels being randomly selected. The hemodynamic quantities were all quantified over the entire mouse brain. For all analyses, we calculated the percentage changes from their baseline values. We used paired *t* tests, and all statistical data were presented with a standard error of the mean (SEM), with *P* values of <0.05 being considered significant.

## Supplementary information

Supplementary Information

The polygon scanner working in air and water

Supplementary Video 2. Mouse brain hemodynamic response to hypoxia challenge

Supplementary Video 3. Close-up hemodynamic response to hypoxia challenge

Supplementary Video 4. Mouse brain hemodynamic response to SNP

Supplementary Video 5. Close-up hemodynamic response to SNP

Supplementary Video 6. Mouse brain hemodynamic response to ischemic stroke

Supplementary Video 7. Close-up hemodynamic response to ischemic stroke

The vasoconstriction during a propagating SD wave

The sO2 change during a propagating SD wave

The originating position of the five SD waves
