# Supplementary material for: Real-time whole-brain imaging of hemodynamics and oxygenation at micro-vessel resolution with ultrafast wide-field photoacoustic microscopy
Source: Light Sci Appl. 2022 May 17;11:138. doi: 10.1038/s41377-022-00836-2 (PMC9110749; doi:10.1038/s41377-022-00836-2)
Supplement: Supplementary file 1 — Supplementary Information [file 41377_2022_836_MOESM1_ESM.docx]

**Supplementary Information for ‘Real-time whole-brain imaging of hemodynamics and oxygenation at micro-vessel resolution with ultrafast wide-field photoacoustic microscopy’**

Xiaoyi Zhu^1^, Qiang Huang^1,5^, Anthony DiSpirito^1^, Tri Vu^1^, Qiangzhou Rong^1^, Xiaorui Peng^1^, Huaxin Sheng^3^, Xiling Shen^1^, Qifa Zhou^2,3^, Laiming Jiang^2,3,*^, Ulrike Hoffmann^4,*^, Junjie Yao^1,*^

^1^ Department of Biomedical Engineering, Duke University, Durham, NC 27708, USA

^2^ Department of Biomedical Engineering, University of Southern California, Los Angeles, CA 90089, USA

^3^ Roski Eye Institute, Department of Ophthalmology, Keck School of Medicine, University of Southern California, Los Angeles, California 90033, USA.

^4^ Department of Anesthesiology, Duke University, Durham, NC 27708, USA

^5^ Department of Pediatric Surgery, Second Affiliated Hospital of Xi’an Jiaotong University, Xi’an, Shaanxi, China

^*^Correspondence: [laiming_jiang@foxmail.com](mailto:laiming_jiang@foxmail.com) for ultrasound transducer; [ulrike.hoffmann@duke.edu](mailto:ulrike.hoffmann@duke.edu) for the small animal studies; [junjie.yao@duke.edu](mailto:junjie.yao@duke.edu) for the overall project

**
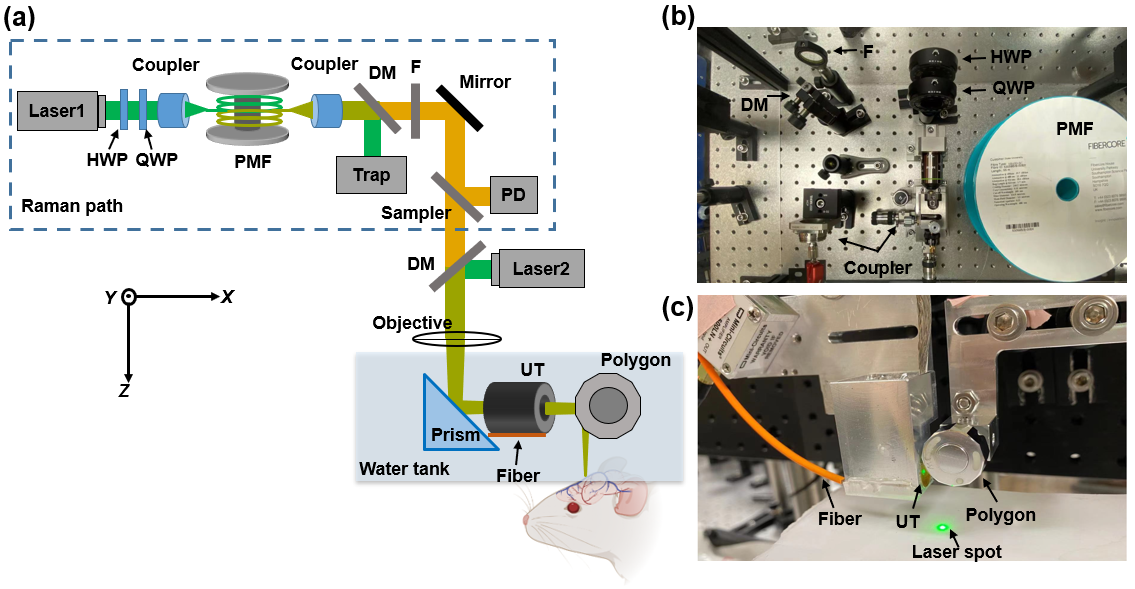
**

**Supplementary Figure 1. The UFF-PAM system**. (a) The detailed schematic of UFF-PAM system. HWP, half-wave plate; QWP, quarter-wave plate; PMF, polarization maintaining fiber; DM, dichroic mirror; F, filter; PD, photodiode; UT, ultrasound transducer. (b) The photo of Raman-shifter system. (c) The photo of polygon scanning system.


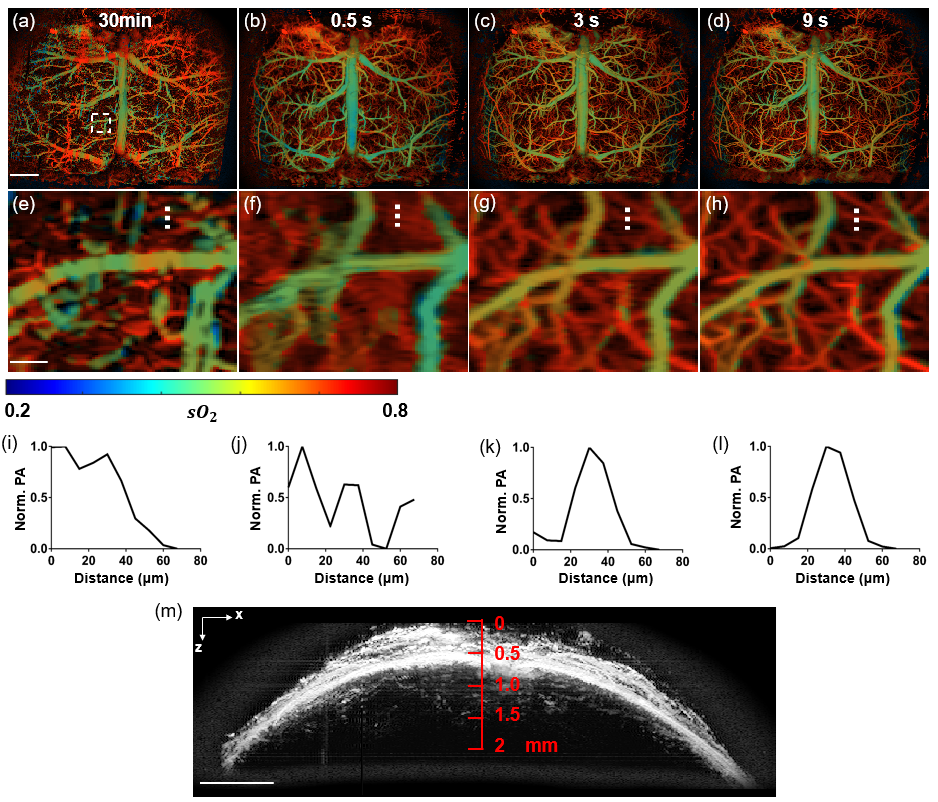


**Supplementary Figure 2. UFF-PAM of the brain vasculature with different imaging speeds**. (a) The sO_2_ image captured by the slow scanning mode with a total imaging time of 30 min to mimic the traditional PAM. Here, we intentionally fixed the mouse head loosely to exaggerate the motion artifacts. The slow scanning result shows the ‘wavy vessels’ caused by breathing motion artifacts. (b-d) The sO_2_ images captured on the same mouse by the fast scanning mode with a total imaging time of 0.5 s, 3 s, and 9 s. (e-h) Close-up images of the dashed box regions from (a-d). (i-l) The signal intensity profiles of a representative vessel marked by the white dotted lines in (e-h), respectively. (m) Representative enhanced x-z MAP image of brain vasculature without depth scanning in the same brain shown in (a-d). Scale bar, 1mm for (a-d, i), 100 μm for (e-h). The results show that the imaging speed of UFF-PAM can be improved at the price of image quality.


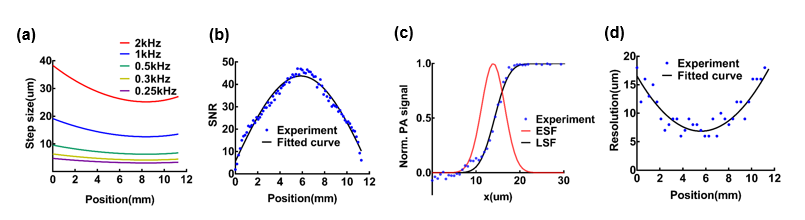


**Supplementary Fig. 3. Imagin**g **parameters of UFF-PAM.** (a) Calculated step sizes at different scanning positions and different scanning speeds. (b) The signal to noise ratio (SNR) of a black tape at different scanning positions. (c) Measured edge spread function (ESF) and derived line spread function (LSF) at the middle of the scanning range, showing a lateral resolution of 7 μm. (d) Measured lateral resolutions at different scanning positions.


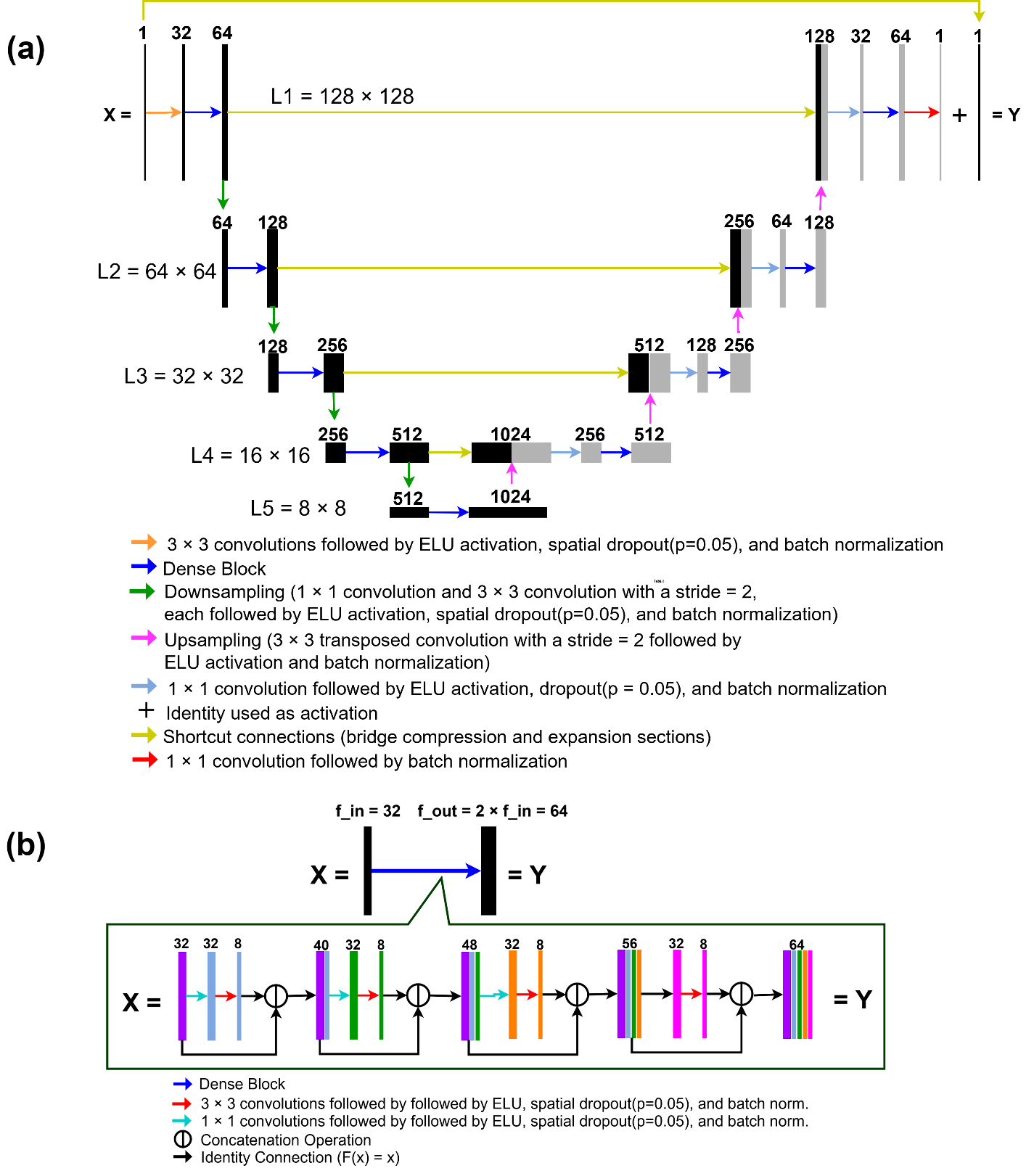


**Supplementary Fig. 4** **The deep learning model**. (a) FD U-net Architecture (b) FD U-net Dense Block. Adapted from DiSpirito et al. [25].


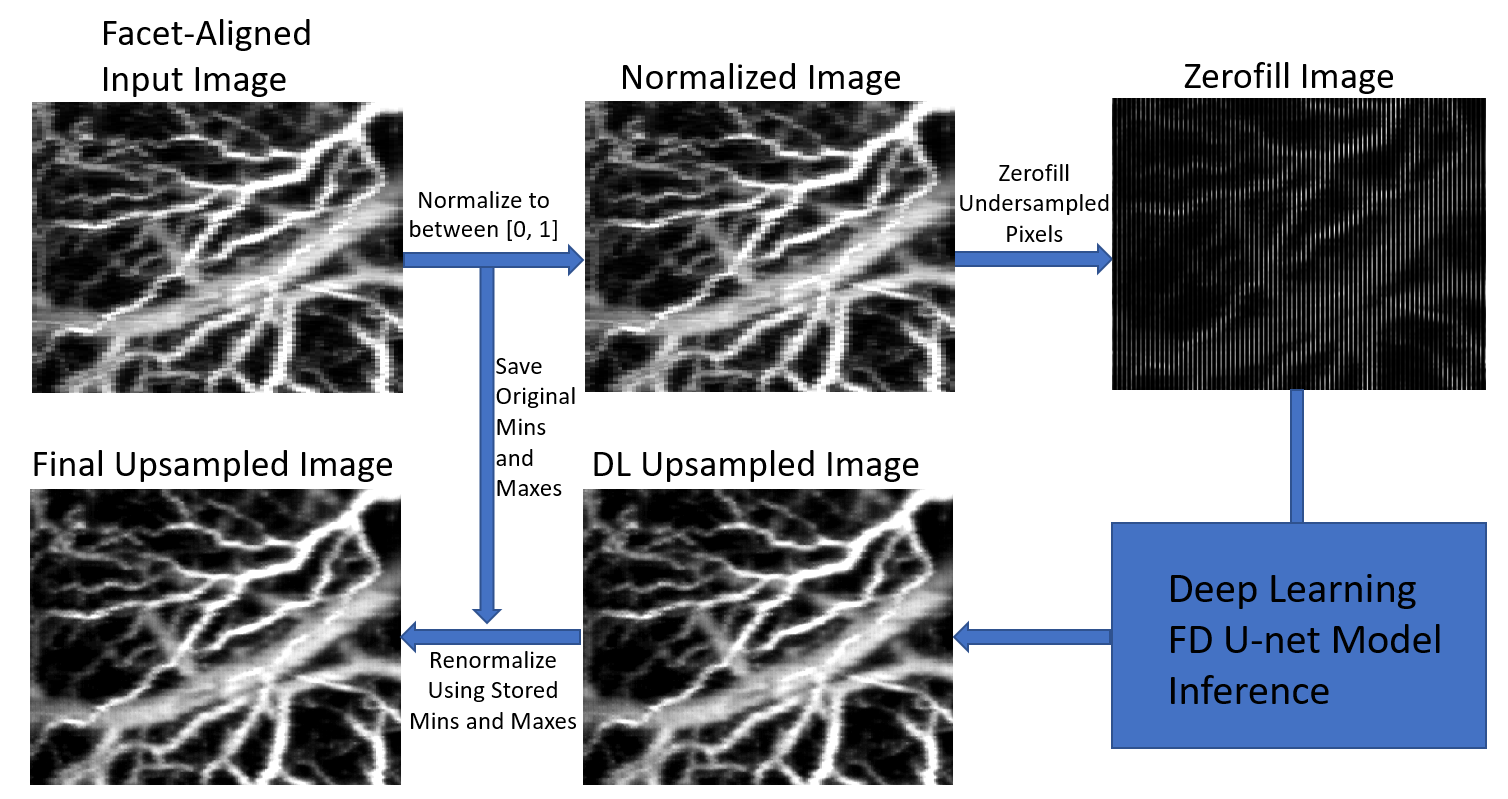


**Supplementary Fig. 5 The image processing pipeline for deep learning image upsampling.** Depicts image upsampling process of image normalization (with saving of mins maxes^-1^), zerofill, FD U-net deep learning model inference, and final renormalization (using saved mins maxes^-1^).


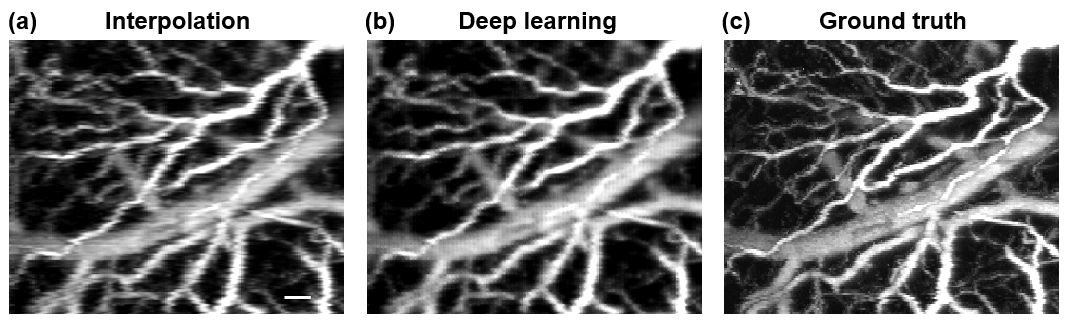


**Supplementary Figure 6. Comparison of the bicubic interpolation and the deep learning based upsampling result.** (a) The bicubic interpolation result. (b) The deep learning based upsampling result. Bicubic interpolation creates reconstructions with jagged vessel boundaries, while the deep learning method does not. (c) The ground truth image.


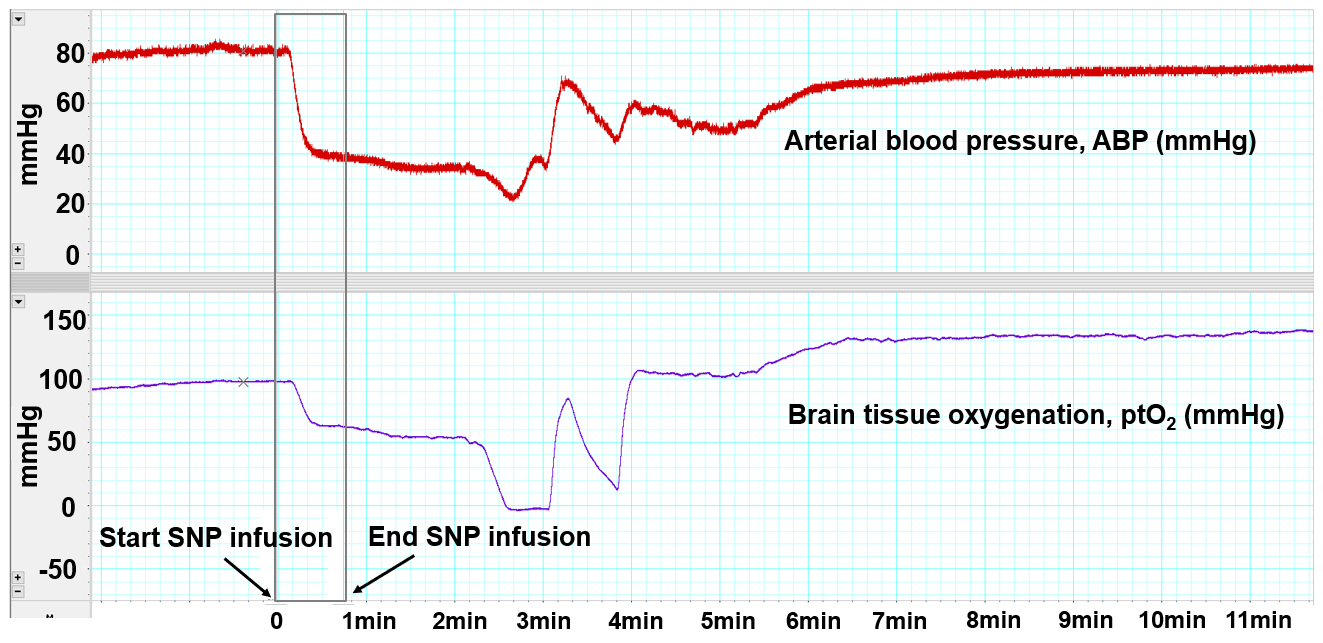


**Supplementary Figure 7. SNP induced changes in arterial blood pressure (ABP) and tissue oxygenation (ptO_2_).** SNP infusion (grey outline) resulted in a substantial decrease in arterial blood pressure from 80 mmHg to 30 mmHg, and consecutive decrease in brain tissue oxygenation. After the SNP infusion, blood pressure spontaneously recovered and oxygenation in the tissue was restored within 5 minutes (n=2).


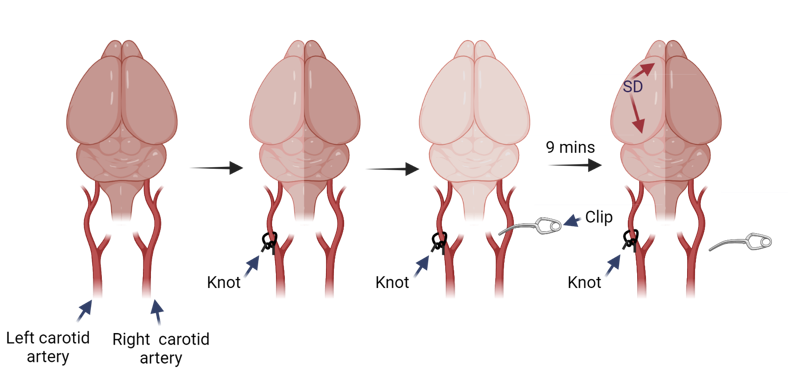


**Supplementary Figure 8. The surgery procedure for the ischemic stroke with permanent occlusion to the left carotid artery and temporary occlusion to the right carotid.**


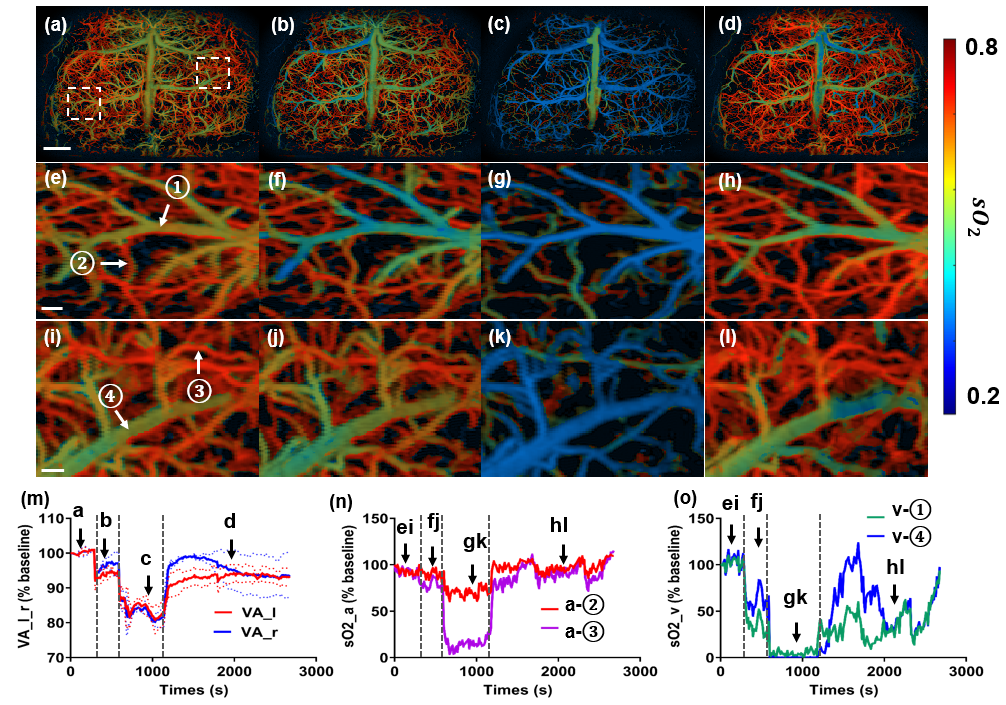


**Supplementary Figure 9. UFF-PAM of the ischemic stroke and restoration in the mouse brain.** (a) The baseline sO_2_ image. (b) The sO_2_ image after permanently ligating the left carotid artery. (c) The sO_2_ image 5 min after temporally clamping the right carotid artery and inducing ischemic stroke. (d) The sO_2_ image 25 min after removing the clamp, showing a sudden restoration of blood flow. (e-h) and (i-l) Close-up images at the same time points of (a-d), indicated by the left (e-h) or right (i-l) white dotted rectangles in (a). (m) Chane in vessel area in the left hemisphere (VA_l) and the right hemisphere (VA_r). (n) The sO_2_ changes of artery ② in the left hemisphere and ③ in the right hemisphere. (o) The sO_2_ changes of vein ① in the left hemisphere and ④ in the right hemisphere. The three vertical gray dotted lines represent the time points of permanent ligation of the left carotid artery, temporally clamping the right carotid artery, and removing the clamp, respectively. The arrows in (m-o) represent the time points of (a-d). N=3. Scale bar, 1 mm for (a-d), 100 μm for (e-l).

**Supplementary Table 1. Summary of UFF-PAM parameters for all the figures**

| **Imaging Parameters** | **Fig. S2a** | **Fig. S2b** | **Fig. S2d** | **Fig.1c,d; Fig.2a,b,f; Fig.3a-c; Fig.4 a-d; Fig.5 a,d.**  **Fig. S2c**  **Fig. S9a-d** | **Fig. 1e** | **Fig.2c-e** | **Fig.3d-f** | **Fig.4e-h** | **Fig.5e, f** |
| --- | --- | --- | --- | --- | --- | --- | --- | --- | --- |
| **Field of view (mm^2^)** | 8 ×7.5 | 8 ×7.5 | 8 ×7.5 | 8 ×7.5 | 8 × 3 | 1.2×1.5 | 1.1 × 1.2 | 1.15 × 1.2 | 0.87 × 0.9 |
| ***x* step size (Polygon, µm)** | 5 | 26-38 | 7-10 | 15-19 | 15-19 | 15-19 | 15-19 | 15-19 | 15-19 |
| ***y* step size (motor, µm)** | 5 | 10 | 1.25 | 2.5 | 2.5 | 2.5 | 2.5 | 2.5 | 2.5 |
| ***x* scanning range (mm)** | 8 | 11 | 11 | 11 | 11 | 11 | 11 | 11 | 11 |
| **1D rate (kHz)** | 1 | 800 | 800 | 800 | 800 | 800 | 800 | 800 | 800 |
| **2D rate (Hz)** | 1 | 2000 | 667 | 1000 | 1000 | 1000 | 1000 | 1000 | 1000 |
| **3D rate (Hz)** | 0.00056 | 2 | 0.11 | 0.33 | 0.33 | 0.33 | 0.33 | 0.33 | 0.33 |
| **Pulse energy (532nm) (nJ)** | 200 | 200 | 200 | 200 | 200 | 200 | 200 | 200 | 200 |
| **Pulse energy (558nm) (nJ)** | 200 | 200 | 200 | 200 | 200 | 200 | 200 | 200 | 200 |

**Supplementary Table 2. Additional UFF-PAM parameters for the three in vivo experiments**

| **Parameters** | **Hypoxia challenge** | **SNP** | **Ischemic stroke** |
| --- | --- | --- | --- |
| **Total imaging time** | 30 min | 35 min | 45 min |
| **Total image frames** | 600 | 700 | 900 |
| **3D frame rate** | 0.33 Hz | 0.33 Hz | 0.33 Hz |
| **Continuous scanning** | Yes | Yes | No (After 5 min baseline scanning, the mouse was removed for surgery. After the surgery, the subsequent scanning was continuous) |
| **Surgery** | No | Yes. (A catheter was inserted into the mouse vein for measuring blood pressure) | Yes (See Fig. S8) |

**Supplementary Movie Captions.**

**Supplementary Movie 1. The polygon scanner working in air and water**

The diamond-ground metal polygon with 12 facets is driven by a water-immersible high-speed DC motor. It can steer light in both air and water. When it scans in water, it can simultaneously steer the confocally aligned laser excitation and ultrasound detection.

**Supplementary Movie 2. Mouse brain hemodynamic response to hypoxia challenge**

UFF-PAM of the entire mouse cortex under hypoxia. We applied systemic hypoxia to the animals by decreasing the breathing oxygen content from 21% (normoxia) to 3% (hypoxia). After baseline imaging with normoxia, we introduced hypoxia for 2 minutes and imaged the resultant hemodynamic changes in the brain vasculature. We repeated the normoxia-hypoxia cycle three times. Left panel: the PA images of the vasculature acquired at 532 nm. Right panel: the sO_2_ images acquired from the dual-wavelength measurements.

**Supplementary Movie 3. Close-up hemodynamic response to hypoxia challenge**

Close-up images of the area indicated by the dashed box in **Fig. 3(a)**. Left panel: the PA images of the vasculature acquired at 532 nm. Right panel: the sO_2_ images acquired from the dual-wavelength measurements.

**Supplementary Movie 4. Mouse brain hemodynamic response to SNP**

UFF-PAM of the entire cortex hemodynamics in response to SNP. After monitoring the brain for 5 mins as the baseline, the SNP was infused via a femoral vein catheter over 40 seconds. Left panel: the PA images of the vasculature acquired at 532 nm. Right panel: the sO_2_ images acquired from the dual-wavelength measurements.

**Supplementary Movie 5. Close-up hemodynamic response to SNP**

Close-up images of the area indicated by the dashed box region in **Fig .4(a)**. Left panel: the PA images of the vasculature acquired at 532 nm. Right panel: the sO_2_ images acquired from the dual-wavelength measurements.

**Supplementary Movie 6. Mouse brain hemodynamic response to ischemic stroke**

We imaged the entire cortex for 5 minutes as the baseline, 5 minutes after permanent ligation of the left carotid artery, and 9 minutes after temporally clamping the right carotid artery to cause bilateral occlusion and induce severe ischemic stroke, and another 26 min after removing the clamp. Left panel: the PA images of the vasculature acquired at 532 nm. Right panel: the sO_2_ images acquired from the dual-wavelength measurements.

**Supplementary Movie 7. Close-up hemodynamic response to ischemic stroke**

Close-up images of the area indicated by the dashed box in **Fig. S9a**. Left panel: the PA images of the vasculature acquired at 532 nm. Right panel: the sO_2_ images acquired from the dual-wavelength measurements.

**Supplementary Movie 8. The vasoconstriction during a propagating SD wave**

The PA signal intensity at 532 nm decreased as a vessel constricted during the SD wave propagation. The change in PA signal intensity is encoded in red on top of the original vasculature image.

**Supplementary Movie 9. The sO_2_ change during a propagating SD wave**

The spreading SD wave of vasoconstriction was accompanied by local hypoxia. This change in sO_2_ is encoded in yellow on top of the original sO_2_ image.

**Supplementary Movie 10. The originating position of the five SD waves**

The close-up images of the originating position of the five SD waves, showing the vasoconstriction and local hypoxia of two representative vessels.
